# Lipoteichoic acids from *Staphylococcus aureus* stimulate proliferation of human non-small-cell lung cancer cells in vitro

**DOI:** 10.1007/s00262-017-1980-4

**Published:** 2017-03-17

**Authors:** Katja Hattar, Christian P. Reinert, Ulf Sibelius, Mira Y. Gökyildirim, Florentine S. B. Subtil, Jochen Wilhelm, Bastian Eul, Gabriele Dahlem, Friedrich Grimminger, Werner Seeger, Ulrich Grandel

**Affiliations:** 1 Department of Internal Medicine IV/V, University of Giessen and Marburg Lung Center (UGMLC), Klinikstrasse 33, Giessen, Germany; 2 Department of Radiotherapy and Radiooncology, Philipps-University, Marburg, Germany; 3 Department of Internal Medicine II, University of Giessen and Marburg Lung Center (UGMLC), Giessen, Germany; 4 Max-Planck Institute for Heart and Lung Research, Bad Nauheim, Germany; 5 Asklepios Klinik Lich, Lich, Germany

**Keywords:** Lung cancer, Infection, Lipoteichoic acids, Tumor proliferation, Interleukin-8, Toll-like-receptor-2

## Abstract

Pulmonary infections are frequent complications in lung cancer and may worsen its outcome and survival. Inflammatory mediators are suspected to promote tumor growth in non-small-cell lung cancer (NSCLC). Hence, bacterial pathogens may affect lung cancer growth by activation of inflammatory signalling. Against this background, we investigated the effect of purified lipoteichoic acids (LTA) of *Staphylococcus aureus* (*S. aureus*) on cellular proliferation and liberation of interleukin (IL)-8 in the NSCLC cell lines A549 and H226. A549 as well as H226 cells constitutively expressed TLR-2 mRNA. Even in low concentrations, LTA induced a prominent increase in cellular proliferation of A549 cells as quantified by automatic cell counting. In parallel, metabolic activity of A549 cells was enhanced. The increase in proliferation was accompanied by an increase in IL-8 mRNA expression and a dose- and time-dependent release of IL-8. Cellular proliferation as well as the release of IL-8 was dependent on specific ligation of TLR-2. Interestingly, targeting IL-8 by neutralizing antibodies completely abolished the LTA-induced proliferation of A549 cells. The pro-proliferative effect of LTA could also be reproduced in the squamous NSCLC cell line H226. In summary, LTA of *S. aureus* induced proliferation of NSCLC cell lines of adeno- and squamous cell carcinoma origin. Ligation of TLR-2 followed by auto- or paracrine signalling by endogenously synthesized IL-8 is centrally involved in LTA-induced tumor cell proliferation. Therefore, pulmonary infections may exert a direct pro-proliferative effect on lung cancer growth.

**Electronic supplementary material:**

The online version of this article (doi:10.1007/s00262-017-1980-4) contains supplementary material, which is available to authorized users.

## Introduction

Lung cancer is the leading cause of cancer-related death in the western hemisphere [1]. In the course of the disease, patients frequently develop pulmonary infections which have been reported to reduce the median survival substantially [2]. It is not clear, whether this reduction in median survival is merely attributable to the clinical complications of pulmonary infections, or whether bacteria may directly stimulate lung cancer growth.

Although the most common pathogens found in NSCLC are of Gram-negative origin, Gram-positive germs such as *Staphylococcus aureus* (*S. aureus*) and *Streptococcus pneumoniae* account for about 25% of pulmonary infections in lung cancer patients and are the leading cause of septicemia in lung cancer [3]. Cell wall components of bacterial pathogens such as lipopolysaccharides, the so-called “endotoxin” of Gram-negative bacteria and their Gram-positive equivalents, lipoteichoic acids (LTA), peptidoglycanes and lipopeptides (Lpp) [4] are major bacterial pathogenicity factors. After ligation of LPS to the CD14 molecule [5], cellular activation is initiated by binding to toll-like receptors. It is widely accepted that TLR-4 confers responsiveness to LPS [6, 7] while TLR-2 seems to be the key receptor for LTA [8–10]. Once TLR-dependent signalling is initiated, a plethora of proinflammatory mediators such as cytokines and lipid mediators are released by immunocompetent cells [8, 11].

It is well established that persistent inflammation and inflammatory mediators can promote cancer growth [12–14]. In lung cancer, a clear pathogenic role has been attributed to chronic inflammatory diseases such as chronic obstructive pulmonary disease [15]. One early step in the development of lung cancer is the activation of inflammatory cascades resulting in synthesis of growth factors and cytokines such as TGF-ß, IL-1, and IL-8 [15]. Once lung cancer has developed, further tumor progression may be caused by inflammatory mediators [16]. Among these inflammatory mediators IL-8 is of special relevance, because in cultured NSCLC cells and in animal models of NSCLC IL-8 has been shown to promote tumor growth [17, 18]. Moreover, in lung cancer patients, there is a clear correlation between IL-8 expression, tumor angiogenesis and overall survival [19].

Synthesis of IL-8 is induced in response to activation of TLRs in myeloid-derived cells such as macrophages and neutrophils [20, 21]. Interestingly, the expression of TLRs is not restricted to myeloid-derived cells. As TLRs are found in a variety of human cancers of epithelial origin, they could definitively play a role in cancer progression. In gastric cancer, the expression of different TLRs enables gastric carcinoma cells to interact with *Helicobacter pylori* [22], which is followed by the production of tumor-promoting factors such as IL-8 [23] and proliferation of cancer cells [24]. Remarkably, an up-regulation of TLR-4 expression was recently demonstrated in human adenocarcinoma of the lung in vivo and TLR-4 expression levels correlated with malignancy [25]. TLR-2 is equally expressed by NSCLC cells in vitro [26] and TLR-2 mRNA has been detected in the bronchoalveolar fluid of patients with NSCLC [27].

Thus, specific interactions between bacterial pathogens and tumor cells may actually occur in NSCLC. For LPS, enhancement of lung cancer tumor growth has been described in NSCLC cell lines and in xenograft and in orthotopic models of lung cancer [28, 29]. In contrast, the consequences of the interaction between lung cancer cells and LTA are less obvious.

In the current study, we investigated the effect of highly purified LTA from *S. aureus* on proliferation and metabolic activity in human NSCLC cell lines of adeno- and squamous cell carcinoma origin. In essence, we found that LTA is a pro-proliferative stimulus for the tumor cell lines. Cellular activation proceeded via ligation of TLR-2 and endogenously formed IL-8 turned out to be a key mediator in NSCLC proliferation induced by LTA.

## Materials and methods

### Cell culture and authentication

The human lung adenocarcinoma cell line A549 (ATCC-CCL-185) as well as the human lung squamous carcinoma cell line H226 were obtained from the American Type Culture Collection (Rockville, MD, USA) and cultured at 37 °C in a humidified atmosphere (95% air, 5% CO_2_). Cells were used up to passage 40. Cells were regularly checked for contamination with mycoplasma by the local department of microbiology by analysis of 16S r DNA followed by amplicon sequencing as previously described [30, 31]. Moreover, both cell lines used were subjected to authentication by the German Collection of Microorganisms and Cell Cultures (“Deutsche Sammlung von Mikroorganismen und Zellkulturen GmbH”, DSMZ) by short-tandem repeat (STR) DNA profiling [32]. STR profiles of the currently used cell lines showed a full match with the respective reference STR profiles. Thus, the A549 and H226 cells used in the current study were derived from authentic cell cultures. All cell culture media and supplements were from Gibco (Eggenstein, Germany), and cell culture plasticware was from Greiner Bio-One (Frickenhausen, Germany). NSCLC cell lines were grown in Dulbecco’s modified Eagle’s medium (DMEM/F12), supplemented with 10% FCS, 2 mM l-glutamine, 10^5^ U/l penicillin and 100 mg/l streptomycin (culture medium). Cells were grown to confluence and subcultured every 2–3 days at a split ratio of 1:10.

### Assessment of cellular proliferation by cell counting

A549 or H226 cells were seeded on 24-well plates (15,000 cells/well for A549 and 30,000 cells/well for H226) and maintained in culture medium for 24 h before stimulation with LTA (*S. aureus*, purified, InvivoGen, San Diego, CA, USA) After removement of culture medium by two washing procedures with RPMI, cells were kept in DMEM/F12 supplemented with 1% FCS at a total volume of 500 µl/well. NSCLC cells were exposed to various concentrations of LTA or sham-incubation was performed (control). In an additional series of experiments in Fig. 4, function-blocking antibodies targeting TLR-2 (clone TL2.1, e-Bioscience, San Diego, CA, USA), TLR-4 (clone HTA 125, e-Bioscience, San Diego, CA, USA), or IL-8 (MAB 208, R&D Systems, Wiesbaden, Germany) were applied simultaneously to LTA. Antibodies targeting TLRs were applied at 0.5 µg/ml, whereas the neutralizing IL-8 antibody was used at 5 µg/ml. At the end of the incubation period from 24 to 72 h, medium was removed, cells were washed twice and subsequently treated with 0.5% trypsin–EDTA. Detached cells were resuspended in a stop-solution (PBS containing 20% FCS) and subsequently counted automatically by the cell counter-analyzer system CasyR Model TT (Innovatis AG, Reutlingen, Germany). Data were expressed as percentage of controls (sham-incubated cells), which were set to 100%. Two technical replicates per sample were run in each independent experiment.

### MTS assay

The MTS assay (CellTiter 96@ Aqueous One Solution Cell Proliferation Assay, Promega, Mannheim, Germany) quantifies the metabolic activity of cells. This assay is used to quantify cellular proliferation and is based upon the cleavage of the yellow 3-(4, 5-dimethylthiazol-2-yl)-5-(3-carboxymethoxyphenyl)-2-(4-sulfophenyl)-2H-tetrazolium, inner salt (MTS) to purple formazan by metabolic active cells. A549 or H226 cells were seeded on 96-well plates (2500 cells/well for A549 and 5000 cells for H226) 24 h before stimulation with LTA in culture medium. After removement of culture medium and two washing procedures, cells were kept in DMEM/F12 supplemented with 1% FCS at a total volume of 200 µl/well. Both NSCLC cell lines were stimulated with increasing concentrations of LTA for various time periods; alternatively, sham-incubated controls were run for each time period. 2.5 h before the end of the incubation period, 20 µl of MTS solution were added to each well and plates were incubated for another 2.5 h under light protection and continuous shaking. Then, absorbance was read at 490 nm, background readings were subtracted from the sample wells and data were expressed as percentage of controls (sham-incubated cells) which were set to 100%. Five technical replicates per sample were run in each independent experiment.

### Measurement of IL-8

IL-8 was quantified from the cell supernatants of LTA-stimulated A549 cells by ELISA technique. For these experiments, A549 cells (15,000 cells/well) were seeded on 24-well plates and grown to confluence. Confluent monolayers were washed twice and kept in DMEM/F12 with 1% FCS at a total volume of 500 µl/well. Then, incubation with different concentrations of LTA or sham-incubation (control) was performed for various time periods. In a separate set of experiments, stimulation with LTA was performed in the absence or presence of antibodies targeting TLR-2 and TLR-4. Two technical replicates per sample were run in each independent experiment. At the end of the incubation period, cell supernatants were harvested, cell debris was removed by centrifugation at 13.000×*g* and samples were stored at −20 °C until further processing. Release of IL-8 was determined in a direct sandwich ELISA, as described previously [33]. To normalize the data, IL-8 was expressed as ΔIL-8, meaning that baseline levels of IL-8 secreted from unstimulated controls were subtracted from those induced by stimulation with LTA.

### RNA isolation and real-time RT-PCR

For quantification of IL-8 mRNA, experiments with A549 cells (50,000 cells/well) were performed as described above. Each independent experiment consisted of two technical replicates per sample.

Total RNA was extracted from cells with TRIzol Reagent (Invitrogen, Karlsruhe, Germany) according to the manufacturer’s protocols. Extracted RNA was quantified with Nano Drop (PeqLab, Erlangen, Germany). Residual DNA was digested with DNase (Invitrogen, Karlsruhe, Germany) and cDNA was synthesized by RT (Bio-Rad, München, Germany). Real-time PCR was performed using 1 μg of cDNA, SYBR Green PCR Master Mix (Bio-Rad, München, Germany) and 0.05 M forward/reverse primers; specific primers used for sequence detection were as follows:

for *IL-8*:

5′AGTTTTGCCAAGGAGTGCTAAA3′ (forward) and 5′TGAATTCTCAGCCCTCTTCAAA3′ (reverse).

for *PBGD*:

5′CAGCTTGCTCGCATACAGAC3′ (forward) and 5′GAATCTTGTCCCCTGTGGTG3′ (reverse).

for *TLR2*:

5′AGCCTTGACCTGTCCAACAA3′ (forward) and 5′GGCTTGAACCAGGAAGACGA3′ (reverse).

Real-time-reactions were carried out on the CFX Connect Real-Time PCR Detection System (Bio-Rad, München, Germany) with following cycle conditions: denaturation at 95 °C for 3 min; 40 cycles with denaturation at 95 °C for 10 s, annealing at 59 °C for 10 s and extension at 72 °C for 10 s. To ensure single-product amplification, a dissociation curve was generated for each gene and the threshold cycle (Ct values) for each gene was determined.

Relative mRNA-levels were expressed as ΔCt values which were calculated with the genes Ct values normalized to the housekeeping gene PBGD Ct values. The comparative 2^ΔΔCt method was used to analyze mRNA-fold changes between control and LTA, which was calculated as ratio = 2^(ΔCt control−ΔCt LTA) [28, 34]. Ct is the cycle threshold and ΔCt (Ct gene of interest−Ct housekeeping gene) is the CT value normalized to the housekeeping gene PBGD obtained for the same cDNA samples. The specificity of the primer pair products was tested by agarose (1.5%) gel electrophoresis (Supplementary Fig. 1).

## Statistics

Unlike otherwise indicated, data are given as the relative changes compared to control values and expressed as the mean ± SEM. Data analysis was performed in R [35] using the packages “lme4” and “lmerTest” [36]. Data were analyzed with linear mixed models to account for inter-experimental differences. The variable “time” was taken as a factor in a two-factorial model together with “LTA concentration”. Data were checked for the agreement with the model assumptions by analysis of the residuals. MTS activity data were inversely transformed before statistical analysis to meet the model assumptions. The diagrams show the means with SEM. The horizontal dashed line indicates the value of the unstimulated controls. For statistical analysis of changes in mRNA expression, student´s *t* test was performed. Groups or conditions with *p* < 0.05 are marked with asterisks.

## Results

### A549 and H226 cells express TLR-2 mRNA

To confirm that the NSCLC cell lines used express TLR-2 as a prerequisite for specific interaction with LTA, we analyzed TLR-2 mRNA by PCR. PDGD served as housekeeping gene. Unstimulated A549 and H226 cells were clearly positive for TLR-2 mRNA, as depicted in Table 1a. Interestingly, upon treatment with 0.1 µg/ml LTA for 24 h, TLR-2 expression was doubled in A549 cells, whereas H226 cells did not show any up-regulation of TLR-2 mRNA after stimulation with LTA (Table 1b). Specificity of the primer pairs was visualized by agarose (1.5%) gel electrophoresis (Supplementary Fig. 1).

**Table 1:** (a) Basal expression of TLR-2 mRNA in A549 and H226 cells (*n* = 4), (b) regulation of TLR-2 mRNA expression upon stimulation with LTA in A549 and H226 cells, *n* = 4

(a)	A549, Ct	H226, Ct
TLR-2 mRNA	27.69 ± 0.43	27.70 ± 0.39
PBGD mRNA	21.41± 0.18	21.94 ± 0.13

(b) TLR-2mRNA	A549, fold-regulation	H226, fold regulation
Control	1	1
LTA 0.1 µg/ml	1.99 ± 0.39*	0,90 ± 0.20

* *p* < 0.05 vs control

### LTA induces proliferation of A549 cells

**Fig. 1:**
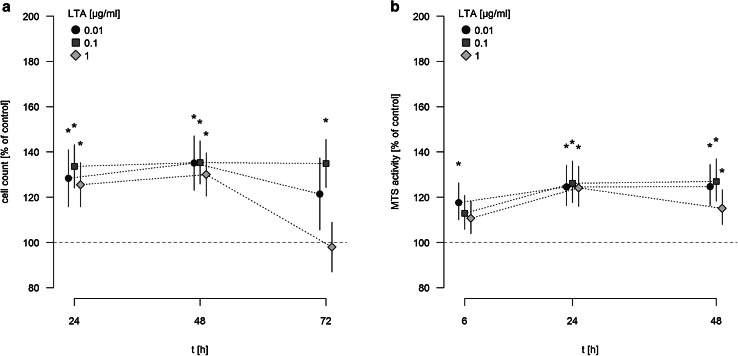
Time-dependent induction of A549 proliferation and MTS activity by LTA. **a** A549 cells were incubated with various concentrations of LTA from *S. aureus* or sham-incubated (control). A549 proliferation was assessed by automatic cell counting. The *horizontal dotted line* indicates the baseline proliferation of sham-incubated cells, which was set to 100%. All data are expressed as percentage of baseline proliferation. Means ± SEM of at least seven independent experiments are given. *Values marked with an asterisk* differ significantly from controls (*p* < 0.05). **b** A549 cells were incubated with various concentrations of LTA from *S. aureus* or sham-incubated (control). Metabolic activity of A549 cells was quantified by MTS assay. *The horizontal dotted line* indicates the baseline proliferation of sham-incubated cells, which was set to 100%. All data are expressed as percentage of baseline proliferation. Means ± SEM of at least seven independent experiments are given. *Values marked with an asterisk* differ significantly from controls (*p* < 0.05)

A549 cells were stimulated with different concentrations of LTA (0.01–1 µg/ml) for various time periods (24, 48, 72 h) or sham-incubation was performed (baseline). The highly purified LTA preparation stimulated the proliferation of A549 cells in a time-dependent manner, as quantified by automatic cell counting (Fig. 1a). LTA-induced increase in proliferation is expressed as percentage of baseline levels, which was set to 100%. Even low concentrations of LTA (0.01 µg/ml) were capable of inducing a significant increase in cellular proliferation. After 24 and 48 h of stimulation, all LTA concentrations induced increases in proliferation by ~25 to 35% of baseline levels. After 72 h of stimulation, increased proliferation was still observed when low (0.01 µg/ml) concentrations of LTA were used, whereas stimulation with 1 µg/ml LTA over this time period was ineffective. The most prominent increase in cellular proliferation was observed after stimulation with 0.1 µg/ml LTA for 48 h (increase by 35.36% ± 1.25% of baseline levels).

LTA failed to induce any pro-proliferative activity of A549 cells when applied at lower concentrations than 0.01 µg/ml: After 24 h, proliferation rates were 105.1 ± 2.7% of baseline upon stimulation with 0.001 µg/ml LTA and 103.4 ± 2.9% after stimulation with 0.0001 µg/ml LTA (*n* = 5). Also after longer incubation periods with 0.001 µg/ml LTA and below, no significant pro-proliferative effect of LTA was observed (e.g. after 48h, proliferation induced by 0.001 µg/ml LTA was 105.5 ± 0.7 and 99.2 ± 1.9% of baseline levels when 0.0001 µg/ml LTA were used, respectively, *n* = 5).

The assessment of metabolic activity, as quantified by MTS assay, showed a comparable pro-proliferative effect of LTA on A549 cells (Fig. 1b). MTS activity was expressed as percentage of baseline levels, which was set to 100%. After 6 h of incubation a significant increase in MTS activity, was noted after stimulation with 0.01 µg/ml LTA. After longer incubation periods of 24 and 48 h, all LTA concentrations increased metabolic activity of A549 cells by ~20 to 25% of baseline levels. Just as observed by automatic cell counting, the most prominent increase in MTS activity of A549 cells was noted after stimulation by 0.1 µg/ml LTA for 48 h (increase by 26.9 ± 1.8% of baseline levels).

### LTA induces a time- and dose-dependent release of IL-8 in a TLR-2-dependent manner

**Fig. 2:**
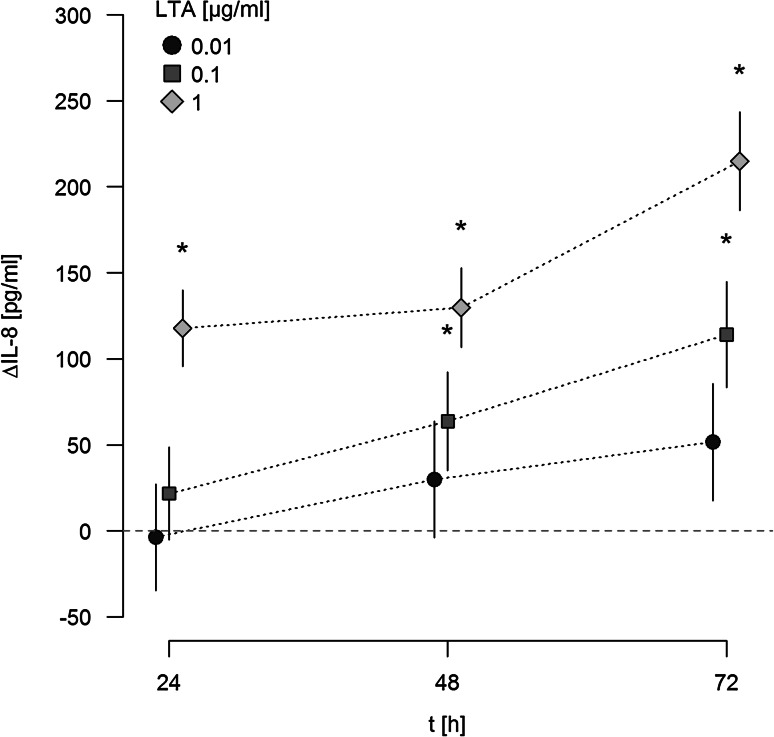
Release of IL-8 from A549 cells in response to LTA. A549 cells were either sham-incubated (control) or exposed to the given concentrations of LTA. At indicated time points, cell supernatants were collected and analyzed for IL-8 by ELISA technique. IL-8 release is given as ΔIL-8, which is the difference between IL-8 released from LTA-stimulated and sham-incubated cells (indicated by the *horizontal dotted line*). ΔIL-8 is expressed in pg/ml. Means ± SEM of at least four independent experiments are given. *Values marked with an asterisk* differ significantly from controls (*p* < 0.05)

When supernatants of LTA-activated A549 cells were analyzed for IL-8 release by ELISA, a dose-dependent release of this chemokine was noted upon stimulation with LTA (Fig. 2). IL-8 release was analyzed after 24, 48, and 72 h, and was expressed as ΔIL-8 versus baseline secretion at indicated time points. Baseline levels of IL-8 liberated from sham-stimulated A549 cells were 53, 98 and 83 pg/ml after 24, 48 and 72 h respectively, and were increased by 117, 129 and 214 pg/ml upon stimulation with 1 µg/ml LTA. In parallel, IL-8 mRNA was upregulated 3.50-fold in response to stimulation with 0.01, 3.54-fold upon stimulation with 0.1 µg/ml, and 11.56-fold in response to 1 µg/ml LTA after 24 h as compared to sham-incubated cells.

**Fig. 3:**
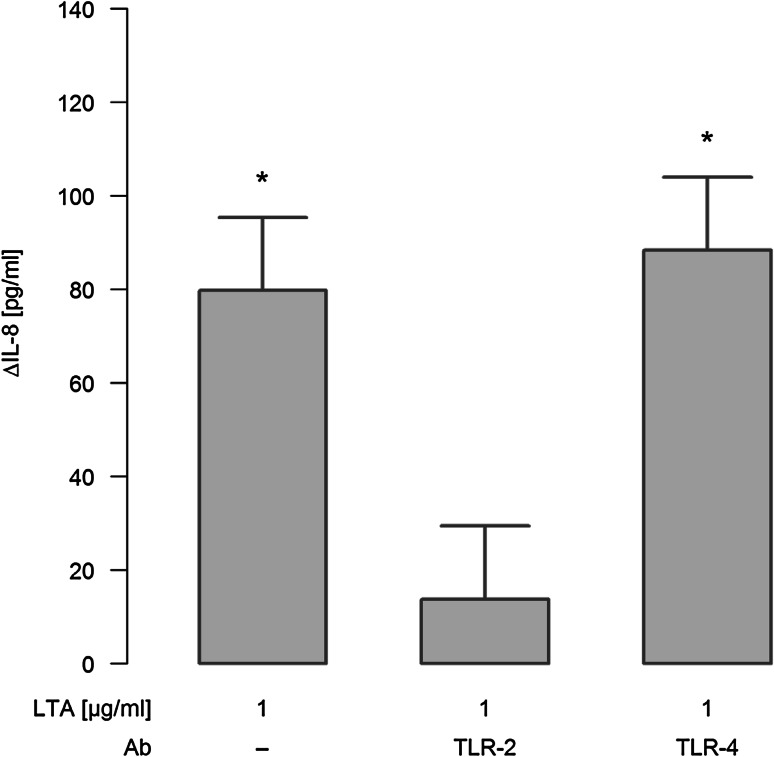
Effect of TLR-2/-4 antagonists on LTA-induced synthesis of IL-8. A549 cells were either sham-incubated (control) or exposed to 1 µg/ml of LTA in the absence or presence of neutralizing antibodies targeting TLR-2 or TLR-4 for 48 h. Release of IL-8 into the cell supernatants was analyzed by ELISA technique and is given as ΔIL-8 (pg/ml), which was calculated by subtracting IL-8 values of sham-incubated cells from LTA-stimulated cells. Data are expressed as means ± SEM of at least six independent experiments. *Values marked with an asterisk* differ significantly from controls (*p* < 0.05)

When ligation of TLR-2 was blocked by neutralizing antibodies, IL-8 release in response to 1 µg/ml LTA was strongly attenuated, as depicted for a stimulation period for 48 h in Fig. 3. In contrast, neutralizing TLR-4 had no inhibitory effect on LTA-induced release of this chemokine.

### Mechanisms of LTA-induced A549 cell proliferation in vitro

**Fig. 4:**
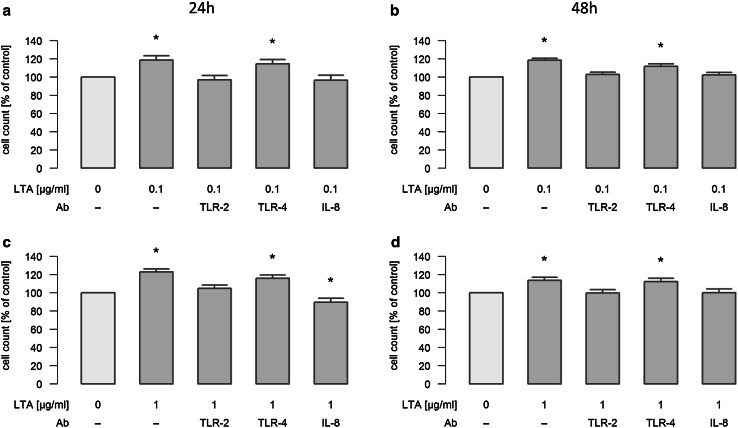
Mechanisms of LTA-induced proliferation of A549 cells. A549 cells were either sham-incubated (control) or exposed to 0.1 (**a**/**b**) or 1 µg/ml (**c**/**d**) of LTA for 24 or 48 h in the absence or presence of neutralizing antibodies targeting TLR-2, TLR-4 or IL-8. A549 proliferation was assessed by automatic cell counting. All data are expressed as percentage of baseline proliferation of sham-incubated cells, which was set to 100%. Means ± SEM of at least four independent experiments are given. *Values marked with an asterisk* differ significantly from controls (*p* < 0.05)

In order to determine the mechanism of LTA-stimulated A549 cellular proliferation, TLR-2, TLR-4 and IL-8 were blocked by antibodies under different experimental conditions. Concretely, stimulation with 0.1 and 1 µg/ml LTA was performed in the absence or presence of these neutralizing antibodies for 24 and 48 h, and cellular proliferation was assessed by automatic cell counting. Stimulation of cellular proliferation induced by 0.1 and 1 µg/ml LTA for 24h was inhibited by interference with TLR-2, whereas inhibition of TLR-4 had no effect (Fig. 4a/c). This inhibitory effect was still reproducible when stimulation with LTA (0.1 and 1 µg/ml) was performed for 48 h (Fig. 4b/d). Most interestingly, neutralization of endogenously produced IL-8 abolished LTA-induced proliferation of A549 cells under all experimental conditions (Fig. 4 a–d). Exogenous IL-8 (2 ng/ml) induced a pro-proliferative response of 126.8 ± 3.7% (*n* = 6) as compared to baseline levels (100%), which was not blocked by the presence of anti-TLR2-antibodies (increase in proliferation to 125.7 ± 3.8%, *n* = 6), nor did the TLR-2 antibodies affect baseline proliferation (baseline proliferation in the presence of TLR-2 antibodies was 100.7 ± 2.2%, *n* = 6).

### The pro-proliferative effects of LTA can be reproduced for the NSCLC squamous carcinoma cell line H226

**Fig. 5:**
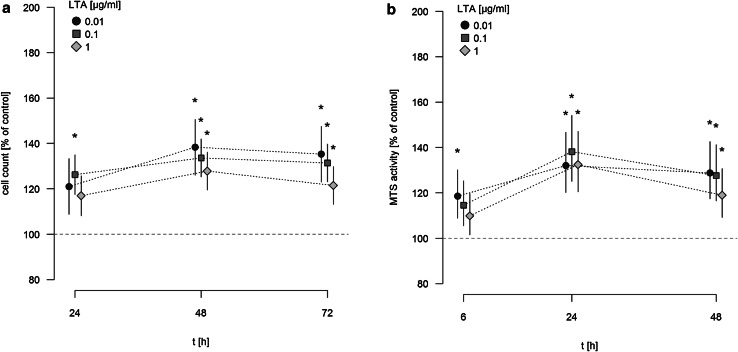
Time-dependent increase in H226 proliferation and MTS activity by LTA. H226 cells were incubated with various concentrations of LTA from *S. aureus* or sham-incubation was performed (control). H226 proliferation was assessed by automatic cell counting (**a**) and metabolic activity was quantified by MTS assay (**b**). The *horizontal dotted line* indicates the baseline proliferation (**a**) and MTS activity (**b**) of sham-incubated cells which was set to 100%. All data are expressed as percentage of baseline proliferation. Means ± SEM of at least four independent experiments are given. *Values marked with an asterisk* differ significantly from controls (*p* < 0.05)

Finally, we tried to reproduce the pro-proliferative effect of LTA in a NSCLC cell line of squamous cell origin. For this purpose, H226 monolayers were stimulated with increasing concentrations of highly purified LTA (0.01, 0.1, and 1 µg/ml) for various time periods (24, 48, 72 h). As assessed by automatic cell counting, LTA induced proliferation of H226 cells approximately to the same extent as previously observed in A549 cells. A strong proliferative response in H226 cells was elicited even by the low LTA-concentration of 0.01 µg/ml. Similar to A549 cells, lower concentrations of LTA did not exert any pro-proliferative effect (data not given). The pro-proliferative effect of LTA was observed over the whole incubation period of 72 h. In parallel, MTS activity was enhanced upon stimulation with LTA, with an early response after 6 h of stimulation and an ongoing increase of MTS activity until 48 h (Fig. 5a/b).

## Discussion

Pulmonary bacterial infections are frequently found in advanced stages of lung cancer and may contribute to the progression of this disease [2, 3]. In this context, bacterial pathogenicity factors may play a decisive role by stimulating cancer cell growth. While a strong pro-proliferative effect has been described for LPS, the endotoxin of Gram-negative bacteria, in lung, liver, ovarian, gastric and breast cancer [28, 29, 37–40], less is known about the pathogenicity factors of Gram-positive germs in this context.

In the present study, we observed that highly purified LTA from *S. aureus* induces proliferation in the lung adenocarcinoma cell line A549. The increase in cellular proliferation assessed by automatic cell counting was paralleled by an enhanced MTS activity. The increase in metabolic activity was clearly related to the increase in cell numbers thus confirming the observation that turnover of MTS is directly proportional to the numbers of viable cells in culture [41]. Notably, in both assays, LTA induced an increase in proliferation by ~30%, thus approaching or even exceeding the biological activity of other well-known endo- or exogenous proliferative agents such as IL-8 [17] or benzo[a]pyrene [42]. The pro-proliferative effect of LTA was not restricted to A549 cells but could be reproduced in a NSCLC line of squamous cell origin, suggesting a pathogenic role of LTA in lung cancer progression in general.

The pro-proliferative effects were clearly caused by LTA and not by contaminating LPS. First, we used highly purified LTA prepared according to the method of Morath et al. [43]. Second, compared to LPS much lower concentrations of LTA (0.01 µg/ml) were sufficient to stimulate cellular proliferation of A549 cells [28, 29]. Third, inhibition of TLR-4 by an antibody that effectively inhibited LPS-induced cellular responses [44, 45], did not affect the LTA-induced proliferation of the NSCLC cell line. And fourth, the LPS-induced pro-proliferative response in A549 cells displayed different kinetics compared to LPS, with a maximum response at 24 h and a rapid decline thereafter [28].

However, we cannot completely rule out that contamination with staphylococcal Lpp may have contributed, at least in part, to the biological activity of the LTA preparation used here. Despite broad evidence of the immunostimulatory potency of LTA [reviewed in 46], Lpp display some immune activation functions by ligation of TLR-2 [26], and some recent reports suggested that the immunostimulatory effects of LTA were, at least in part, mediated by contaminating Lpp [47, 48]. However, synthetic LTA has been demonstrated to activate cytokine synthesis [49] and in a recent report performed in full blood, it has also been shown that binding of LTA to TLR-2 is a prerequisite to elicit cytokine synthesis [50]. Thus, we believe that LTA is a potent activator of cellular inflammatory reactions.

It is noteworthy that, in contrast to LPS-induced stimulation [28, 29], LTA-mediated cellular proliferation displayed no clear dose-dependency. LTA-concentrations from 0.01 to 1 µg/ml induced proliferative responses. This observation is reminiscent of the “all or nothing” principle in human biology, which means that a cellular reaction is initiated as soon as a certain “threshold” concentration of receptor saturation is reached [51]. Therefore, one could speculate that TLR-2 receptors are “saturated” by 0.01 µg/ml LTA, as lower concentrations of LTA failed to induce any significant pro-proliferative response. The absence of a clear dose-dependency in LTA-induced cellular proliferation corresponds well to the studies of Rezania et al., in which increased metabolic activity of human prostate cancer cell lines over a wide range of LTA-concentrations displayed no dose-dependency [10].

When higher concentrations of LTA (1 µg/ml) were used, cellular proliferation could not be further enhanced and was even reduced to baseline levels after the longest incubation period. This may be explained to the previously reported pro-apoptotic effects of LTA in high concentrations which may counteract the pro-proliferative effects of LTA at least in higher concentrations [52].

In our experimental setup LTA induced proliferation of A549 cells by a sequence of TLR-2 ligation, and subsequent activation of IL-8 synthesis. As a prerequisite for specific interaction with LTA, constitutive expression of TLR-2 mRNA was proven in both cell lines by real-time RT-PCR. Interestingly, LTA even induced up-regulation of TLR-2 mRNA in A549 cells. The relevance of TLR-2-expression was clearly demonstrated by the use of function blocking antibodies to TLR-2 and TLR-4. While LTA-induced release of IL-8 was abrogated in the presence of a TLR-2-antibody, blocking of TLR-4 did not affect IL-8 secretion in response to LTA. The specificity of the TLR-2 antibody was proven by the fact that it did not affect baseline or IL-8 induced proliferation of A549 cells. The TLR-2-dependency of LTA-induced proliferation is in line with previous studies which demonstrated that LTA-induced cellular responses proceed via TLR-2 and further down-stream signaling involving pathway-specific TRAFs, activation of NF-κB and subsequent cytokine synthesis [8, 9, 53]. Supporting our findings, it has recently been demonstrated that TLR-2 activation is also operative in human gastric and breast cancer [54].

Clearly, biological activity of IL-8 was a prerequisite for A549 cell proliferation in our study. When IL-8 was inhibited by a neutralizing antibody proliferation of A549 cells in response to LTA was completely abolished. The observation that in the low concentration of LTA (0.01 µg/ml) no significant amounts of IL-8 were detected, does not stand against this mechanism, as there was still a trend towards elevated IL-8 levels. Moreover, IL-8 mRNA was elevated nearly to the same extent whether 0.01 or 0.1 µg/ml LTA were used. We assume that IL-8 is liberated from A549 cells and activates adjacent A549 cells in an auto- or paracrine way without necessarily reaching significant concentrations in the cell supernatants under all experimental conditions.

Auto- and/or paracrine activation of A549 proliferation by IL-8 correspond well to the in-vitro-studies of Luppi et al., which demonstrated that exogenously added IL-8 stimulates proliferation in the NSCLC cell lines [55]. In line with these results, we demonstrated that addition of exogenous IL-8 stimulated A549 proliferation. Although we did not address the exact signaling events in our study, one mechanism of autocrine cell activation may be direct stimulation of the CXCR receptor type 1, which has been shown to be the decisive receptor subtype mediating proliferative responses upon stimulation with IL-8 in A549 cells [56]. Alternatively, IL-8 may activate NSCLC growth by trans-activation of EGFR, one of the key “drivers” of NSCLC, especially when activating mutations are found [57]. The capacity of IL-8 to transactivate EGFR has not only been shown in A549 cells [55], but also in gastric epithelial and endothelial cells [58, 59]. Besides its direct effect of tumor cell activation, IL-8 is a potent pro-angiogenic factor in NSCLC [18, 19], which may be of further relevance in lung cancer patients.

In conclusion, this is the first study which demonstrates that purified LTA of *S. aureus* effectively induces growth of NSCLC cell lines of adeno- and squamous cell carcinoma origin. These effects are mediated by ligation of TLR-2, and IL-8 was identified as a decisive endogenous mediator activating tumor cell growth. Thus, infections with Gram-positive bacteria may directly contribute to tumor growth in lung cancer.

### Electronic supplementary material

Below is the link to the electronic supplementary material.

Supplementary material 1 (PDF 159 KB)

## Abbreviations

DSMZ: Deutsche Sammlung von Mikroorganismen und Zellkulturen GmbH
Lpp: Lipopeptides
LTA: Lipoteichoic acids
MTS: 3-(4, 5-Dimethylthiazol-2-yl)-5-(3-carboxymethoxyphenyl)-2-(4-sulfophenyl)-2H-tetrazolium
NSCLC: Non-small-cell lung cancer
*S. aureus*: *Staphylococcus aureus*
STR: Short-tandem repeat
