# Supplementary material for: Lipoteichoic acids from Staphylococcus aureus stimulate proliferation of human non-small-cell lung cancer cells in vitro
Source: Cancer Immunol Immunother. 2017 Mar 17;66(6):799–809. doi: 10.1007/s00262-017-1980-4 (PMC5445152; doi:10.1007/s00262-017-1980-4)
Supplement: Supplementary file 1 — Supplementary material 1 (PDF 159 KB) [file 262_2017_1980_MOESM1_ESM.pdf]

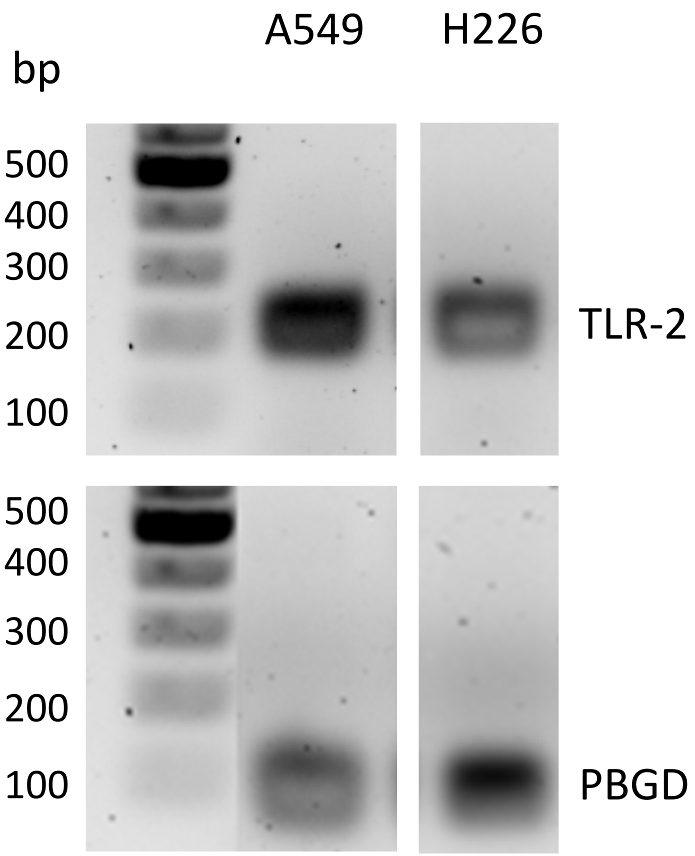

Supplementary figure 1:
TLR-2 expression in A549 and H226 cells
RT-qPCR of TLR-2 and PBGD mRNA was performed. The qPCR amplicon products were run at 100V at 1,5% agarose gel and stained with ethidium bromide. The amplification products were detected at the expected sizes (200bp for TLR-2).

Hattar et al., supplementary figure 1

Cancer Immunology, Immunotherapy (submitted in 2016)
